# Hybrid TiO_2_–Polyaniline Photocatalysts and their Application in Building Gypsum Plasters

**DOI:** 10.3390/ma13071516

**Published:** 2020-03-26

**Authors:** Agnieszka Sulowska, Izabela Wysocka, Daniel Pelczarski, Jakub Karczewski, Anna Zielińska-Jurek

**Affiliations:** 1 Department of Process Engineering and Chemical Technology, Faculty of Chemistry, Gdansk University of Technology, 80-232 Gdansk, Poland; izabela.wysocka@pg.edu.pl; 2 Department of Physics of Electronic Phenomena, Faculty of Applied Physics and Mathematics, Gdansk University of Technology, 80-232 Gdansk, Poland; daniel.pelczarski@pg.edu.pl; 3 Department of Solid State Physics, Faculty of Applied Physics and Mathematics, Gdansk University of Technology, 80-232 Gdansk, Poland; jakub.karczewski@pg.edu.pl

**Keywords:** hybrid nanocomposites, polyaniline, titanium(IV) oxide, phenol, photocatalytic gypsum plaster

## Abstract

Hybrid materials of conjugated polymer and titanium(IV) oxide have attracted considerable attention concerning their potential benefits, including (i) efficient exploitation of visible light, (ii) a high adsorption capacity for organic contaminants, (iii) and effective charge carriers separation. The new class of the photocatalysts is promising for the removal of environmental pollutants in both aqueous and gaseous phases. For the first time, in this study, the polyaniline (PANI)–TiO_2_ hybrid composite was used for the degradation of phenol in water and toluene in the gas phase. Polyaniline–TiO_2_ was prepared by the in situ polymerization of aniline on the TiO_2_ surface. The obtained hybrid material was characterized by diffuse reflectance spectroscopy (DR/UV-Vis), X-ray diffraction (XRD), fast-Fourier transformation spectroscopy (FTIR), photoluminescence (PL) spectroscopy, microscopy analysis (SEM/TEM), and thermogravimetric analysis (TGA). An insight into the mechanism was shown based on the photodegradation analysis of charge carrier scavengers. Polyaniline is an efficient TiO_2_ photosensitizer for photodegradation in visible light (λ > 420 nm). The trapping experiments revealed that mainly h+ and ˙OH were the reactive oxygen species that were responsible for phenol degradation. Furthermore, the PANI–TiO_2_ hybrid nanocomposite was used in gypsum plaster to study the self-cleaning properties of the obtained building material. The effect of PANI–TiO_2_ content on the hydrophilic/hydrophobic properties and crystallographic structure of gypsum was studied. The obtained PANI–TiO_2_-modified gypsum plaster had improved photocatalytic activity in the reaction of toluene degradation under Vis light.

## 1. Introduction

Environmental pollution is one of the 21st-century major threats that negatively affect both human health and the entire ecosystem. Among the currently available techniques for air and water purification, advanced oxidation processes (AOP) are efficient and environment-friendly technologies with a high oxidation potential to complete the degradation of both organic and inorganic contaminants [1]. Among AOPs, heterogeneous photocatalysis is still of growing interest. The basis of photocatalysis consists of the excitation of semiconductors with energy greater than or equal to its bandgap. As a result, the electron is transferred from the valence band to the conduction band, leaving the unoccupied positively charged energy state named hole. Photogenerated charge carriers participate in redox reactions with adsorbed water molecules, oxygen, and hydroxyl ions, leading to the formation of reactive oxygen species that are capable of the non-selective and effective oxidation of impurities [2]. 

The most commonly used photocatalytic semiconductors, including titanium(IV) oxide (TiO_2_) and zinc(II) oxide (ZnO), are activated under ultraviolet radiation, which requires the application of energy-consuming UV lamps, generating higher costs of purification, and limiting the use of green light sources such as sunlight. In this regard, the development of new photocatalysts active under visible light is highly demanded. The most common methods for the modification of semiconductors include (i) surface modification with noble metal nanoparticles [3,4,5,6], (ii) doping with non-metals [7,8,9], and (iii) photosensitization with dyes [10,11]. Recently, conjugated polymers (CPs) have become a new class of semiconductor materials with possible applications in photocatalytic degradation under visible light [12,13].

Hybrid nanomaterials based on the conjugated polymers such as polyaniline (PANI), polythiophene, polypyrrole, or poly(3-hexylthiophene) and inorganic semiconductors can be applied for the degradation of organic pollutants in aqueous phase [12,13,14,15,16]. Among conjugated polymers, polyaniline (PANI) is one of the most commonly used polymers due to its good stability, non-toxicity, low synthesis cost, optical and electrical properties, and high electron mobility [17]. A significant part of the studies related to the conjugated polymer-based photocatalysts are nanocomposites modified with polyaniline (PANI). The PANI was used for the preparation of hybrid composites with the following semiconductors: Ag_2_CO_3_ [18], Ag/Ag_3_PO_4_ [19], Bi_12_O_17_Cl_2_ [20], BiOCl [21], BiPO_4_ [22], BiOI [23], BiOBr [24], BiVO_4_ [25], CoFe_2_O_4_ [26], CoMoO_4_–TiO_2_ [27], Fe_3_O_4_@ZnO [28], MgIn_2_S_4_ [29], MnFe_2_O_4_–N–K_2_Ti_4_O_9_ [30], SnFe_2_O_4_–SnO_2_ [31], Ta_3_N_5_ [32], TiO_2_ [12], TiO_2_@Fe_3_O_4_ [33], V_2_O_5_·nH_2_O [34], ZnO [35], and ZrO_2_ [36]. PANI hybrid nanocomposites are photocatalytic active under visible light due to the HOMO–LUMO (highest occupied molecular orbital- lowest occupied molecular orbital) level excitation and increased separation of electron–hole pairs [12]. A significant part of the research on the photocatalytic activity of PANI–TiO_2_ nanocomposites reported in the literature focused on the photodegradation reaction of dyes e.g., methylene blue [37] and Rhodamine B [38] in the aqueous phase. Moreover, there are only a few studies on the photocatalytic activity of PANI–TiO_2_ nanocomposites in the gas phase reaction [39]. The degradation of pollutants in the gas phase is an important aspect. This approach allows for improving indoor air due to the more effective degradation of xenobiotics in air. 

Furthermore, the hybrid photocatalyst obtained in this study was employed for the preparation of building material with self-cleaning properties. Most of the early studies focused on the addition of titanium(IV) oxide coupled with inorganic additives to obtain self-cleaning building materials [40,41,42]. Janus et al. [42] added a nitrogen and carbon co-modified TiO_2_ photocatalyst to commercial gypsum. The properties of the plaster were determined in RR198 dye degradation, as a model contaminant, under UV and UV-Vis light irradiation. The higher amount of anatase type photocatalysts present in the gypsum matrix increased the self-cleaning ability of the modified gypsum material [42]. A recent study by Gnayem et al. [43] and Singh et al. [44] concluded that the bismuth oxychloride (BiOCl) photocatalyst, which is active in visible light, could be used for the preparation of self-cleaning building material. Singh et al. [44] obtained cementitious containing 1% to 20 wt % BiOCl nanoparticles. The photocatalytic activity was analyzed in the reaction of the Resazurin decomposition under visible light irradiation. The results showed that photocatalytic performance improved linearly up to 5% of BiOCl addition in the cement matrix [44]. In particular, no study, to our knowledge, has considered using conjugated polymer–inorganic semiconductor hybrid nanocomposites in the building materials. 

In this regard, a PANI–TiO_2_ composite was used for the first time as an additive in plaster with photocatalytic properties. In addition, the hydrophilic/hydrophobic properties of gypsum surfaces were measured during contact angle tests. The prepared gypsum samples were irradiated with a wavelength from the UV and visible region to evaluate the photocatalytic activity in the gaseous phase during toluene degradation.

## 2. Materials and Methods 

### 2.1. Materials

Aniline 98% (Sigma Aldrich, Saint. Louis, MO, USA), ammonium persulfate 98% (Sigma Aldrich, Saint. Louis, MO, USA), and hydrochloric acid 36.5% (POCh, Gliwice, Poland) were used as starting materials for the polymerization. As the TiO_2_ matrix, titanium(IV) oxide P25 was supplied by Evonik (Evonik, Essen, Germany). Phenol 99% was purchased from Sigma Aldrich (Sigma Aldrich, Saint. Louis, MO, USA) and selected as a model pollutant. Benzoquinone 98%, ammonium oxalate 99.5%, silver nitrate 99.9%, and tert-butyl alcohol 99.5% were also provided by Sigma Aldrich (Sigma Aldrich, Saint. Louis, MO, USA) and used as scavengers of superoxides, holes, electrons, and hydroxyl radicals, respectively. Acetonitrile (Merck, Darmstadt, Germany) and orthophosphoric acid 85% (VWR, Gdansk, Poland) were used, as eluents, for the HPLC mobile phase. Ethylene glycol 99% was purchased from Sigma Aldrich (Sigma Aldrich, Saint. Louis, MO, USA) and applied for contact angle tests.

### 2.2. Preparation of Photocatalysts

Polyaniline (PANI) was obtained by the oxidative polymerization method. Firstly, 90 cm^3^ of 1 M hydrochloric acid aqueous solution and 0.1 cm^3^ of aniline were mixed in a round-bottomed flask. Then, the oxidant solution was prepared by dissolving 0.25 g of ammonium persulphate in 30 cm^3^ of 1 M hydrochloric acid. The molar ratio of aniline to ammonium persulfate was equal to 1:1. The polymerization reaction was carried out at 0 °C for 24 h. The resulting precipitate was centrifuged, washed three times with water and ethanol, and then dried at 70 °C to constant mass. The potential effect of polymerization on the morphological properties of TiO_2_ and thus the photocatalytic activity was also studied. In this regard, the solution without aniline monomer treated with polymerization mixture was obtained according to the preparation procedure employed for PANI–TiO_2_. To obtain the PANI–TiO_2_ composite, titanium(IV) oxide P25 was added into the acidic aniline solution before oxidation, and oxidative chemical polymerization was performed, as described above. The molar ratio of aniline to TiO_2_ was equal to 1:5.

### 2.3. Preparation of Photocatalytic Gypsum Plaster

The photocatalytic gypsum plaster was prepared by blending commercial gypsum plaster (Dolina Nidy), an addition of photocatalyst, and distilled water in a quantity sufficient to obtain a paste form. The proportions of the individual ingredients were 1.5 g of gypsum, 0.15 g of photocatalyst, and 0.9 cm^3^ of distilled water. Two types of photocatalysts were used: the commercial TiO_2_ (Evonik Aeroxide^®^ TiO_2_ P25) and PANI-TiO_2_ in 10 wt % to the dry mass of the plaster. The blended gypsum was placed in a mold (30 mm diameter; 5 mm height) and dried at room temperature.

### 2.4. Characterization Techniques

The chemical structure of samples was analyzed using an FTIR Nicolet iS10 (Thermo Fisher Scientific Waltham, MA, USA) spectrometer at room temperature in the wavenumber range from 4000 to 400 cm^−1^. Each sample was scanned 64 times at a resolution of 4 cm^−1^. The pellets for FTIR spectroscopy contained 95 wt % of potassium bromide and 5 wt % of a photocatalyst.

XRD analyses were performed using the Rigaku Intelligent X-ray diffraction system SmartLab (Rigaku Corporation, Tokyo, Japan) equipped with a sealed tube X-ray generator (a copper target; operated at 40 kV and 30 mA). Data were collected in the 2θ range of 5°–80°. The scan speed and scan steps were 1° min^−1^ and 0.01°, respectively. The analysis was based on the International Centre for Diffraction Data (ICDD) database. The crystallite size of the photocatalysts in the vertical direction to the corresponding lattice plane was determined using Scherrer’s equation. Quantitative analysis, including phase composition with standard deviation, from the most intensive independent peak of each phase, was performed based on the ICDD database, using the Reference Intensity Ratio (RIR) method.

Nitrogen adsorption–desorption isotherms (BET method for the specific surface area) were measured using the Micromeritics Gemini V (model 2365) (Norcross, GA, USA) instrument at 77 K (liquid nitrogen temperature).

Diffuse reflectance spectra (DR) in the range of 300–800 nm were measured using a ThemoScientific Evolution 220 Spectrophotometer (Waltham, MA, USA) equipped with a PIN-757 integrating sphere. For the measurements, barium sulfate was used as a reference. The photocatalyst samples were mixed with barium sulfate in the proportion of 1:10. The bandgap energy of the photocatalysts was calculated from the corresponding Kubelka–Munk function, $F{\left(R\right)}^{0.5}E_{ph}^{0.5}$ against $E_{ph}$, where $E_{ph}$ is the photon energy. The Tauc transformation was used to determine the bandgap energy (Eg) at the intersection of the straight-line fit of the region associated with the optical absorption edge.

The thermal stability of TiO_2_, PANI, and the PANI–TiO_2_ nanocomposite were studied with thermogravimetric analysis (TGA), using an SDT Q600 V20.9 Build 20 analyzer (New Castle, DE, USA). A sample of 7.5 mg was heated under a nitrogen atmosphere from 28 to 680 °C, with a heating rate of 50 °C·min^−1^. The nitrogen flow rate in TGA was 5 cm^3^·min^−1^.

The morphology of the PANI–TiO_2_ nanocomposite was studied using microscopy analysis on a Tecnai F20 X-Twin (Fei Europe) microscope operated at 200 kV and Cs-corrected operating in transmission and scanning mode. For both scanning and transmission modes, bright field detection was applied. For image processing and fast Fourier transformation (FFT) analysis, ImageJ (Research Services Branch, National Institute of Mental Health, Bethesda, MD, USA) software was used. The morphology of the samples was observed under an FEI Quanta 250 FEG scanning electron microscope (SEM) equipped with an Apollo-X SDD energy-dispersive spectrometer (EDS) with the energy resolution of 134 eV and PEELS (Parallel Electron Energy Loss Spectrometer) with the energy resolution of 0.8 eV, RTEM SN9577+. The EDS data were analyzed using the standard-less analysis in the EDAX TEAM™ (AMETEK Materials Analysis Division, Mahwah, NJ, USA, ver. 4.5) software. For microscopy characterization, the photocatalyst powder was dispersed in ethanol in an ultrasound batch for 1 min. Furthermore, a few drops of the suspension were deposited on a copper mesh coated with a carbon film, and the solvent was evaporated at room temperature. 

The photoluminescence (PL) spectra were recorded on a Perkin-Elmer LS 55 fluorescence spectrometer with a Xenon discharge lamp as the excitation source. The samples were excited at 250 nm in the air at room temperature. A 290 nm cut-off filter was used during measurements. The PL spectra were recorded in the range of 300 to 700 nm. 

Cyclic voltammetry (CV) measurement was carried out using an AutoLab PGStat 302N potentiostat–galvanostat system (Methrom, Autolab) in the standard three-electrode setup. A glassy carbon electrode covered with a thin film of polyaniline was used as a working electrode; Ag/AgCl was used as a reference electrode. CV experiments were performed at a scan rate of 50 mV/s at room temperature. As an electrolyte, a solution of 0.2 M of K_2_SO_4_ and pH equal to 2.8 (by adding H_2_SO_4_) was used.

The contact angle test, by the sessile drop technique, was carried out to evaluate the hydrophilic/hydrophobic properties of the plaster surface. The analysis was performed using the Contact Angle System OCA 25 Instrument (DataPhysics, Filderstadt, Germany). A pellet of gypsum plaster (diameter of 7 mm, the height of 4 mm) was prepared for the contact angle test. On the surface of the pellet, a 12 µL droplet of liquid was placed, and the images were taken. Drops were dispensed automatically at medium speed using a 1 mL syringe. Ethylene glycol was used as a wetting liquid. The results of the contact angle were an average of six measurements.

### 2.5. Photocatalytic Activity

The photocatalytic activity was evaluated in a model reaction of phenol degradation under both UV-Vis and Vis light. As an irradiation source, a 300 W Xenon lamp (LOT Oriel, Darmstadt, Germany) was used. For the visible light measurements, cut-off GG400 and GG420 filters (Optel, Opole, Poland) were used to obtain settled irradiation range above 400 nm and 420 nm, respectively. A photocatalyst at the content of 2 g·dm^−3^ was added to 25 cm^3^ of 20 mg·dm^−3^ phenol solution. The obtained suspension was kept in darkness for 30 min under continuous stirring to provide adsorption–desorption stabilization. The irradiation intensity was controlled using an optical power meter Hioki 3664 (Hioki E.R. Corporation, Nagano, Japan). The light flux in the range of 310–380 nm was equal to 30 mW·cm^−2^. The temperature of the reaction solution was kept at 20 °C using a water bath. Aliquots of 1.0 cm^3^ of the reaction suspension were collected every 20 min and filtered through syringe filters (φ = 0.2 µm) for the removal of photocatalyst particles. The irradiation time was 60 minutes. Phenol and intermediates were determined using a reversed-phase high-performance liquid chromatography (HPLC) system, which was equipped with a C18 chromatography column with bound residual silane groups (Phenomenex, model 00F-4435-E0) and a DAD detector (diode array detector) (model SPD-M20A, Shimadzu). The tests were carried out at 45 °C and under isocratic flow conditions of 0.3 cm^3^·min^−1^ and a volume composition of the mobile phase of 70% acetonitrile, 29.5% water, and 0.5% orthophosphoric acid. Qualitative and quantitative analyses were performed based on previously made measurements of relevant substance standards and using the method of an external calibration curve.

The effect of charge carrier scavengers was examined by addition into the phenol solution (before the introduction of the photocatalyst) of 1 cm^3^ of 500 mg·dm^−3^ of tert-butyl alcohol (t-BuOH), silver nitrate (AgNO_3_), ammonium oxalate (AO), or benzoquinone (BQ), as hydroxyl radicals, superoxide, electron scavengers, and hole scavengers, respectively.

Photocatalytic activity in the gaseous phase was analyzed within the toluene decomposition reaction. A test of toluene photocatalytic decomposition in a flat stainless steel reactor, with a working volume of 30 cm^3^, was performed. The reactor was equipped with a quartz window, two valves, and a septum. The source of radiation was a set of 25 light emitting diodes (LEDs) with an emission range of λ = 365–395 nm, maximum emission at λ_max_ = 380 nm, λ = 380–420 nm, maximum emission at λ_max_ = 400 nm, λ = 430–490 nm, and maximum emission at λ_max_ = 460 nm. The intensity of the incident radiation was measured by a Hioki 3664 meter with a Hioki 9741 sensor. For LEDs with an emission range λ = 365–395 nm, the radiation flux at 380 nm was 2.5 mW·cm^−2^; for λ = 380–420 nm, the radiation flux at 400 nm was 0.63 mW cm^−2^; for the emission range λ = 430–490 nm, the radiation intensity at 460 nm was equal to 24.2 mW·cm^−2^. A mixture of toluene and air for the toluene degradation in the gas phase was prepared in a gas cylinder. The exact volume of toluene (230 µL) was injected into the gas cylinder. Then, the cylinder was filled with air to reach a pressure of 10 bar. The mixture of air and toluene in the gas cylinder was left for 2 days to stabilize. In a typical experiment, a glass plate (20 × 20 mm) covered with photocatalyst or a gypsum–photocatalyst composite film was placed in the center of the reactor. Next, the reactor was filled with the toluene/air mixture at a flow rate of 6 SCFH (standard cubic feet per hour) for 3 min. The outlet of the reactor was connected with a scrubber filled with methanol. The photocatalytic analyses in the gas phase were performed under atmospheric pressure, and the excess of the mixture was passed through the scrubber to prevent the toluene emission to the atmosphere. Then, the outlet and inlet valves were closed. Before irradiation, the system was kept in the dark to achieve adsorption equilibrium. The concentration of toluene was measured chromatographically by collecting gas samples from the reactor through the septum using a gastight syringe. The irradiation time was 3 h, and the samples were collected every 30 min. The toluene concentration was determined using a gas chromatograph (Clarus 500, PerkinElmer) equipped with a flame ionization detector (FID) and DB-1 capillary column (30 m × 0.32 mm, film thickness 3.0 μm). The samples (0.1 cm^3^) were dosed by a gas-tight syringe (Hamilton). Hydrogen at the flow of 1.2 cm^3^·min^−1^, as the carrier gas, was used. 

## 3. Results

### 3.1. Characterization of Photocatalysts

**Figure 1:**
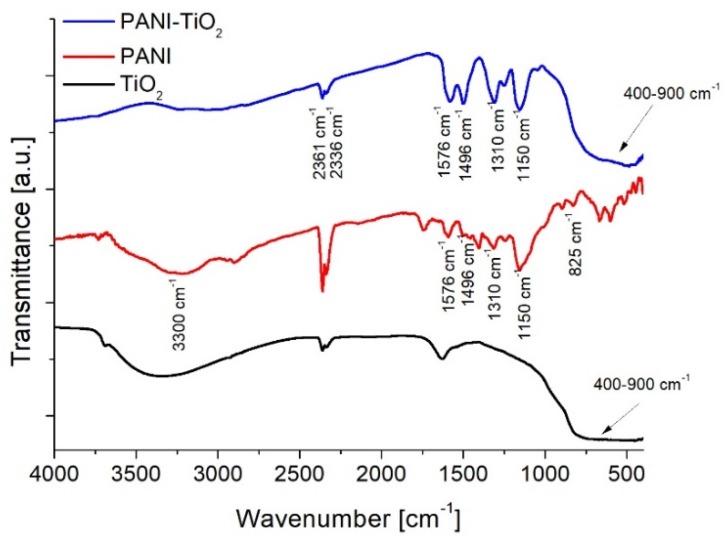
Fast-Fourier transformation spectroscopy (FTIR) spectra of TiO_2_, polyaniline (PANI), and PANI–TiO_2_.

Figure 1 presented the FTIR spectra of PANI, PANI–TiO_2_, and TiO_2_ samples. In the spectra, characteristic peaks attributed to both PANI and TiO_2_ were observed. The characteristic peak for polyaniline at about 3300 cm^−1^ corresponds to the stretching vibrations of the N–H groups. The band at 1576 cm^−1^ corresponds to the stretching vibrations of C=N bonds in the quinone unit. Peaks at 1496 cm^−1^ and 825 cm^−1^ represent stretching vibrations of C=C bonds and C–H in the benzene ring, respectively. Peaks at 1307 cm^−1^ and 1240 cm^−1^ result from C–N bond vibrations. The fast-Fourier transformation spectroscopy (FTIR) spectrum for unmodified titanium(IV) oxide includes an intense and wide peak in the range of 400–900 cm^−1^, which corresponds to Ti–O vibrations in the crystal structure. A wide band in the range of 2900–3600 cm^−1^ corresponds to O–H stretching vibrations of hydroxyl groups. A peak at 1620 cm^−1^ represents bending vibrations of O–H hydroxyl groups resulting from the presence of water adsorbed on the semiconductor surface [2]. Peaks at 2361 cm^−1^ and 2336 cm^−1^ correspond to vibrations of carbon(IV) oxide origin from atmospheric CO_2._

**Figure 2:**
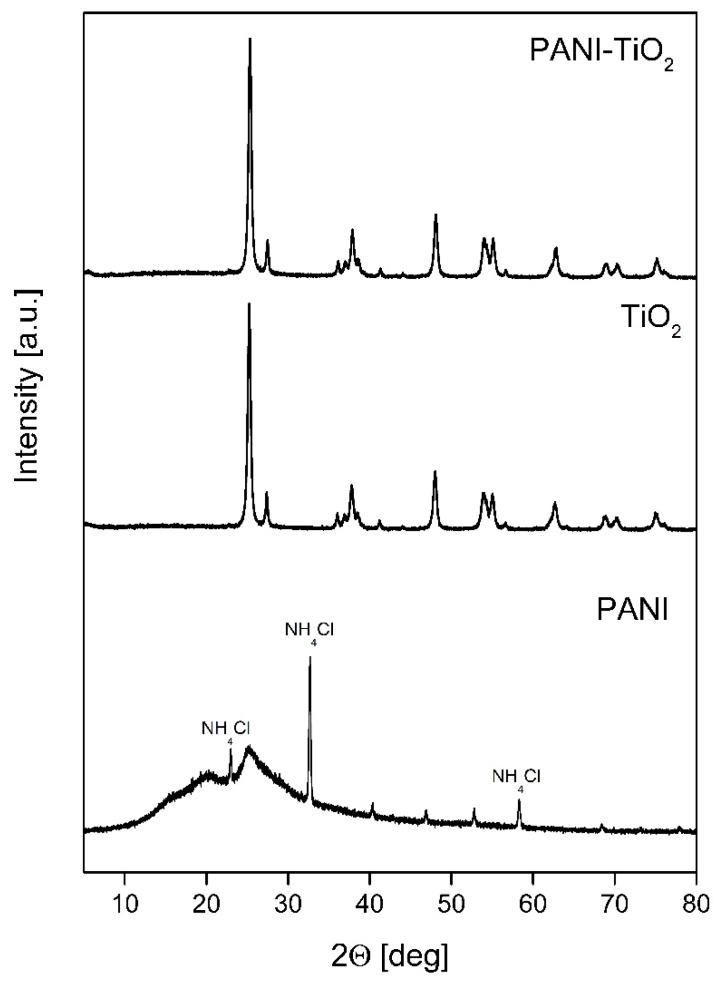
XRD patterns of PANI, TiO_2_, and PANI–TiO_2_.

XRD patterns of PANI, TiO_2_, and PAN–ITiO_2_ are shown in Figure 2. The average crystallite size and values of Brunauer–Emmett–Teller (BET) surface area for TiO_2_ and PANI-TiO_2_ are presented in Table 1. As was expected for commercial TiO_2_ P25, two TiO_2_ crystal phases of anatase and rutile were distinguished. For both TiO_2_ bare and modified with PANI, the crystallite size for anatase and rutile was equal to 18 nm and approximately 25 nm, respectively. The phase content was also similar for PANI–TiO_2_ and TiO_2_. The anatase content was equal to 86.3° ± 0.3° and 81.3° ± 0.9° for TiO_2_ and PANI–TiO_2_, respectively, while the rutile phase fluctuated from 12.9° ± 0.2° to 13.7° ± 0.2°. The reflections at 20.17 ± 0.04° and 24.90° ± 0.06° for PANI indicated the amorphous form of polyaniline. In addition, for PANI, diffractogram peaks at 22.96°, 32.66°, 40.38°, 46.91°, 52.82°, and 58.28° corresponded to the salammoniac, whose presence is caused by polyaniline protonation with hydrochloric acid. The addition of polyaniline in the composite did not change the location and shape of TiO_2_ diffraction peaks. The BET surface, presented in Table 1, did not change after the deposition of polymer particles and was equal to both photocatalysts to 55 m^2^·g^−1^. The BET surface area for pure PANI was 11 m^2^·g^−1^.

**Figure 3:**
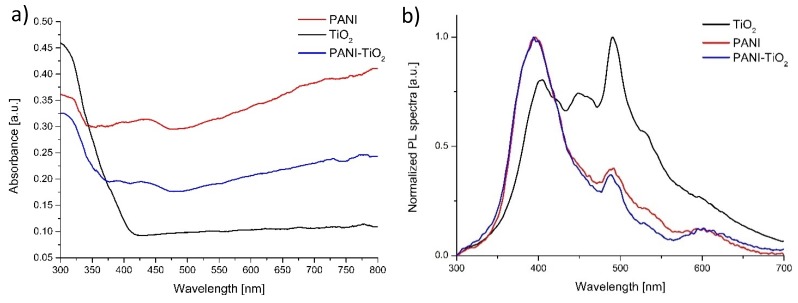
(**a**) Diffuse reflectance spectroscopy (DR/UV-Vis) absorption spectra and (**b**) photoluminescence spectra of pure TiO_2_, PANI, and PANI–TiO_2_ nanoparticles.

The UV-Vis absorption spectrum of pure TiO_2_, PANI, and the PANI–TiO_2_ nanocomposite is presented in Figure 3a. The bare TiO_2_ absorbed only UV light in the range of 200–400 nm, whereas PANI and PANI–TiO_2_ revealed absorption both in the UV and visible light. Polyaniline and the PANI–TiO_2_ hybrid nanocomposite showed stronger absorption in the visible region compared to pure TiO_2_. However, for the PANI–TiO_2_ nanocomposite, we observe a decrease in absorption intensity compared to polyaniline. A signal at around 440 nm for both PANI and PANI–TiO_2_ was observed, which indicated the polaron–π* transition of the quinoid ring [45]. The Tauc transformation of the DR/UV-Vis spectra (see Figure S1) allows determining the optical bandgap energies of TiO_2_ (3.10 eV), PANI (3.37 eV), and PANI/TiO_2_ (3.26 eV) photocatalysts. 

The photoluminescence spectra of TiO_2_, PANI, and the PANI–TiO_2_ nanocomposite were presented in Figure 3b. All samples were at 250 nm wavelength excited. Three different maximum emission signals, in the PL spectra, at 405, 449, and 490 nm were identified for pure TiO_2_. The peak at 3.06 eV (405 nm) corresponded to strong excitonic emission. The emission at 2.76 eV (449 nm) resulted from the presence of trap levels caused by O_2_ vacancies [46]. Furthermore, the peak at 2.53 eV (490 nm) is related to the emission from TiO_2_ surface states [46]. All the emissions are ascribed to the surface emissions and are associated with the trapped charge carriers’ recombination. In the UV range, pure TiO_2_ has a lower intensity, which indicates a lower electron–hole recombination rate and higher photocatalytic activity than for PANI and the PANI/TiO_2_ composite. The signal at 495–530 nm corresponds to O^2−^ vacancies. Therefore, the visible luminescence band for TiO_2_ originates from the oxygen vacancies associated with Ti^3+^ in the TiO_2_ structure. The polyaniline PL spectrum is corresponding to the PL spectra presented in the literature [47,48]. Emission from the UV region, with the maximum at 395 nm, could be attributed to the π*–π transition of the benzoic units in the conjugated polymer. In the visible region, two emission peaks at 487 nm and 605 nm were observed. The peak at 487 nm is related to d-excitation from the polaron band. Rohom et al. [47] also detected a similar emission peak at 613 nm; however, its origin is not clarified. In the visible light region, the intensity of the PL signal was lower for PANI and PANI/TiO_2_ materials compared to TiO_2_ P25. It indicates that PANI can act as an efficient electron donor and hole transported upon visible light (495 nm) radiation. The PL spectrum of the PANI–TiO_2_ hybrid nanocomposite is similar to the emission peak of polyaniline. This observation indicates that the presence of the conjugated polymer affects the light absorption properties of the nanocomposite.

**Figure 4:**
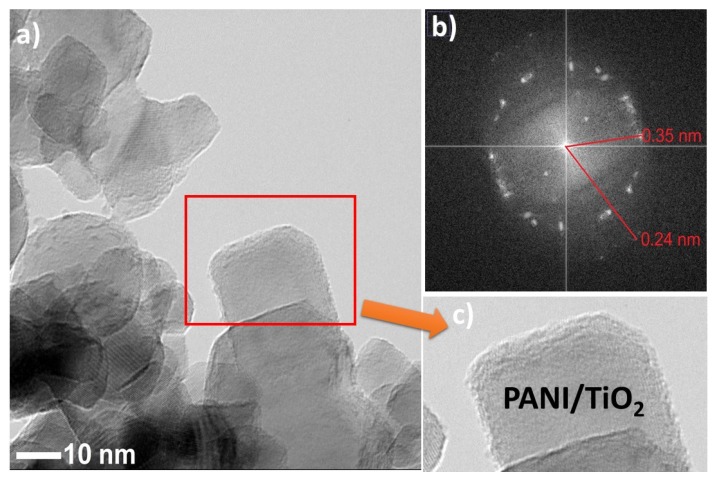
(**a**) TEM image of PANI–TiO_2_; (**b**) FFT analysis of PANI–TiO_2_ nanocomposite; (**c**) magnification on PANI–TiO_2_ structure.

An in-depth observation of the as-prepared samples by TEM analysis was conducted (see Figure 4a–c, and Figure S2). Titanium(IV) oxide particles represented regular thin nanosheets of lamellar shape. Polyaniline was deposited on titanium(IV) oxide edges. Further, FFT (fast Fourier transform) analysis for selected areas was performed. Reflections of d spacing (see in Figure 4b) were equal to 0.35 nm and 0.24 nm, corresponding to anatase TiO_2_ (110) and (202), respectively. The magnification of TiO_2_ particles in Figure 4c showed that the surface is coated with the amorphous layer, which could be derived from polyaniline.

**Figure 5:**
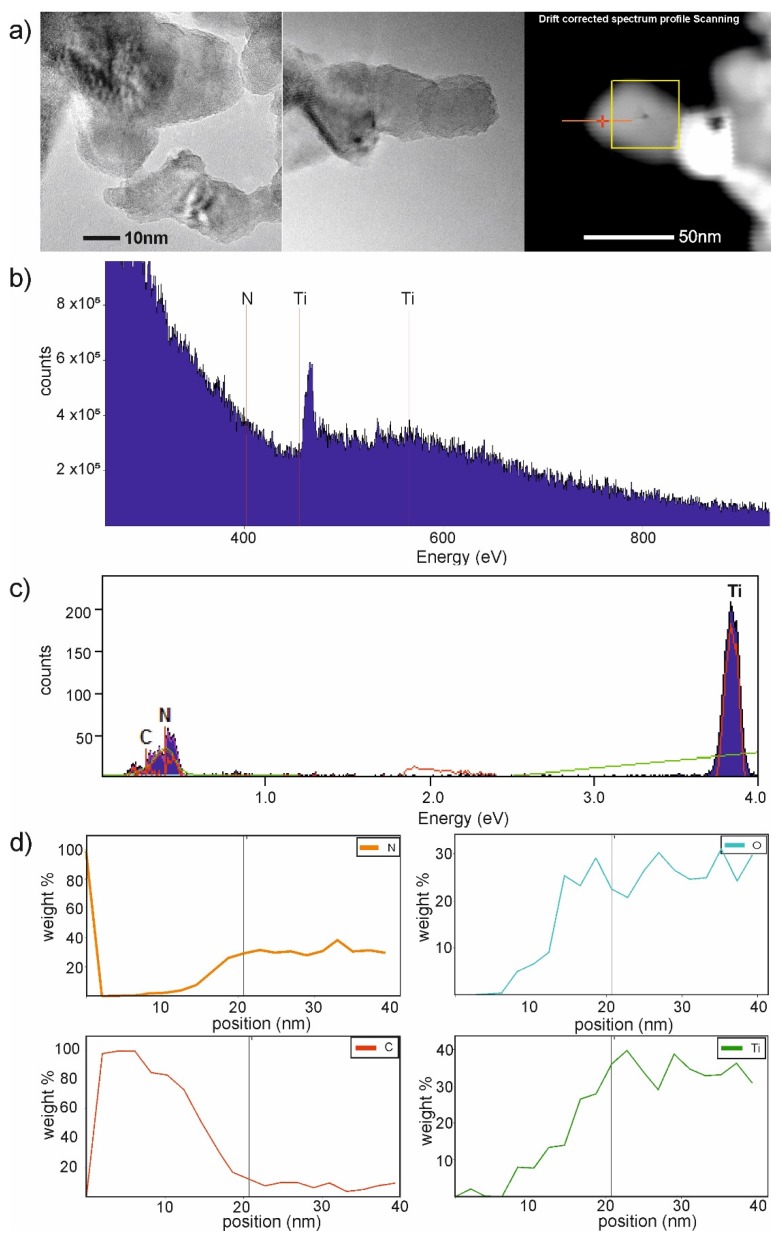
(**a**) TEM images with marked EDS-line area, (**b**–**d**): Results of energy-dispersive spectrometer (EDS)-line measurements and element composition for the PANI–TiO_2_ composite.

Furthermore, the EDS analysis for PANI–TiO_2_ was performed. The results are shown in Figure 5. The presence of carbon and nitrogen was noticed on the PANI–TiO_2_ nanocomposite surface, which may result from modification of the surface of TiO_2_ with polyaniline (C_6_H_8_N_2_). Based on EDS analysis (Figure 5b), the presence of carbon, nitrogen, titania, and oxygen was confirmed. Moreover, the carbon-to-nitrogen ratio (85% C, 15% N) was similar to that in the polyaniline molecule (79% C, 16 % N, and 5% H). The higher carbon content is a result of the background signal from the copper mesh coated with a carbon film gathered outside the sample (see in Figure 5a (right) and Figure 5d). The presence of titania, nitrogen, and oxygen was noticed in the deeper area of the EDS (Energy-dispersive spectroscopy) profile (above 6 nm) toward the sample. 

**Figure 6:**
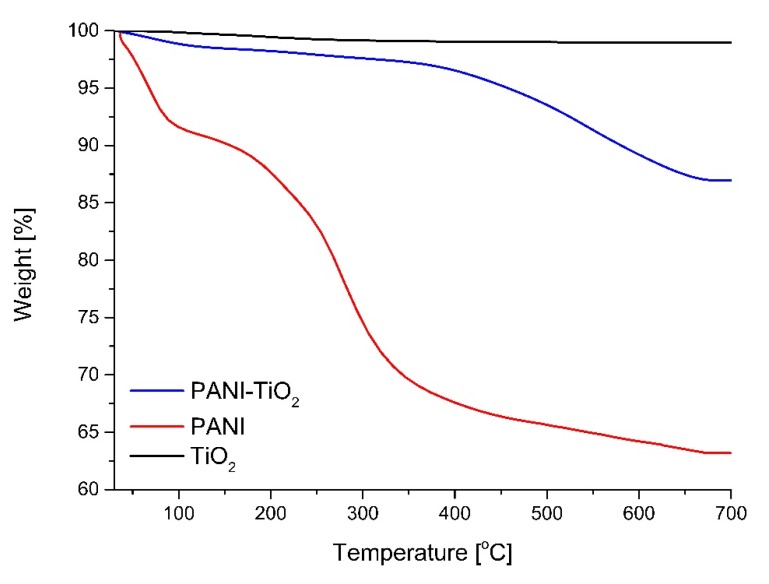
Thermogravimetric analysis (TGA) curve of TiO_2_, PANI, and PANI–TiO_2_ nanocomposite.

The thermal stability of TiO_2_, PANI, and the PANI–TiO_2_ nanocomposite using thermogravimetric analysis (TGA) was analyzed (see Figure 6). For TiO_2_, the weight loss was 1.04%, which is related to the volatilization of H_2_O. Polyaniline revealed a two-step thermal degradation curve. The weight loss up to 54 °C is also related to the volatilization of H_2_O. The first step of thermal degradation is approximately 4% at 80 °C, and the second step is approximately 31% at 650 °C. For PANI–TiO_2,_ first weight loss, at the region up to 300 °C, is attributed to the volatilization of H_2_O and HCl residuals. The second region of sharp weight loss from 350 to 675 °C indicated the breakdown of polyaniline chains [49,50,51]. Above 675 °C, the weight of the PANI–TiO_2_ nanocomposite remained stable, indicating the complete decomposition of polyaniline. The final weight reduction of about 15% corresponded to theoretical polyaniline content in the hybrid nanocomposite.

**Figure 7:**
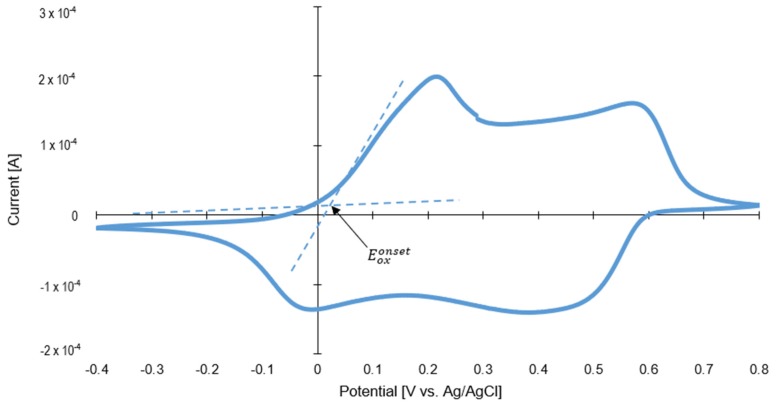
Cyclic voltammograms of polyaniline.

Furthermore, cyclic voltammetry was used (see in Figure 7) to estimate the HOMO level of polyaniline. The energy of the HOMO level was determined from the onset of oxidation potential by taking the E_HOMO_ of ferrocene (equal to 4.8 eV) as a reference value. The E_ox_ of ferrocene versus Ag/AgCl internal standard was equal to 0.48 V. The HOMO energy level was calculated using the following Bredas et al. [52] empirical equations:

(1) $$E_{HOMO}=-4.8\;-[(E_{ox}^{onset}\;-E_{1/2\;ferrocene})]\;\mathrm{eV}$$

(2) $$E_{HOMO}=-4.8\;-[(0.02-0.48)]\;\mathrm{eV}=-4.34\;\mathrm{eV}.$$

Based on the Tauc transformation of DR/UV-Vis spectra (see Figure S1, Supporting Materials), the bandgap for polyaniline is equal to 2.25 eV. The LUMO level of polyaniline was calculated using the following equation:

(3) $$E_{LUMO}=E_{HOMO}+E_{g}=-4.34\;\mathrm{eV}+3.37\;\mathrm{eV}=-0.97\;\mathrm{eV}.$$

### 3.2. Photocatalytic Activity

**Figure 8:**
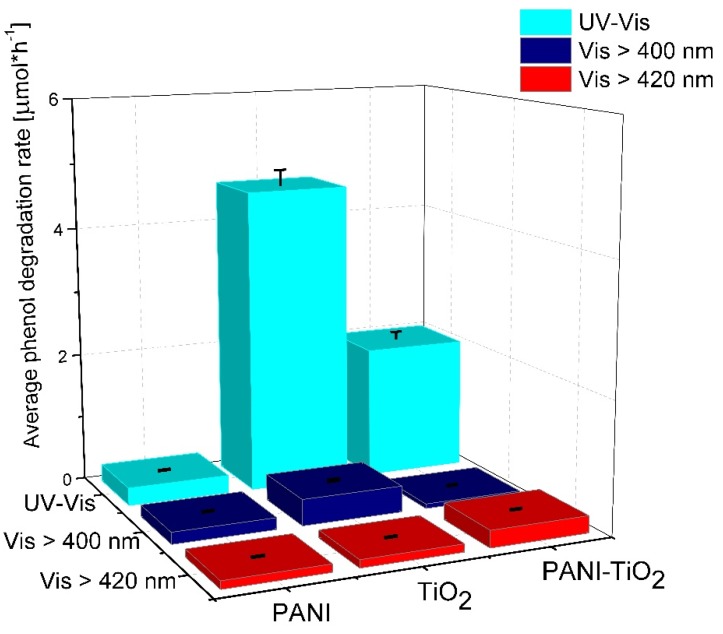
Phenol degradation of pure TiO_2_, PANI, and PANI–TiO_2_ nanocomposite under UV-Vis, Vis (>400 nm) and Vis (>420 nm). Irradiation time: 60 min; photocatalysts loading: 2 g·dm^−3^; phenol initial concentration: 20 mg·dm^−3^; irradiation source: xenon lamp with cut-off filters.

To evaluate PANI–TiO_2_ photocatalytic properties under UV-Vis and visible light degradation of phenol were studied. For visible-light experiments, two cut-off filters, GG400 (λ > 400 nm) and GG420 (λ > 420 nm), were used. The adsorption process proceeded for 30 min in the dark to obtain adsorption/desorption equilibrium conditions. The results of photocatalytic activity for PANI, TiO_2_, and PANI–TiO_2_ nanocomposites in phenol degradation are presented in Table 2 and Figure 8, as well as in Figures S3–S5 in the Supporting Materials. Bare TiO_2_ revealed the highest photocatalytic activity under UV-Vis light among all examined photocatalysts. Additionally, a reference sample of TiO_2_ photocatalyst treated with potassium persulfate and hydrochloric acid (the polymerization mixture) in the absence of aniline was prepared and analyzed in the reaction of phenol degradation to ensure that the polymerization reaction did not influence the TiO_2_ structure. The efficiency of phenol degradation was similar to that for untreated TiO_2_. Based on the results, it was confirmed that the polymerization reaction did not influence the TiO_2_ structure.

Bulk polyaniline exhibited low photocatalytic activity under UV-Vis light. The phenol degradation rate for the PANI–TiO_2_ nanocomposite was equal to 2.01 ± 0.10 µmol·h^−1^ under UV-Vis irradiation. The deposition of PANI particles on the TiO_2_ surface reduced the photocatalytic activity of TiO_2_ under UV-Vis light. PANI particles may play the role of an inner filter, blocking the UV absorption of TiO_2_. Such an effect was observed in our previous results when the photocatalytic activity of TiO_2_ modified with noble metals nanoparticles was lower than bare TiO_2_ [53,54]. On the other hand, polyaniline enhanced the photocatalytic activity of TiO_2_ under a wavelength longer than 420 nm. The phenol degradation rate for the PANI–TiO_2_ nanocomposite under visible light (λ > 420 nm) was equal to 0.26 ± 0.01 µmol·h^−1^. Based on these results, PANI acts as a photosensitizer in the hybrid nanocomposite. Under visible light irradiation up to 400 nm (λ > 400 nm), PANI–TiO_2_ exhibited lower activity in phenol degradation compared to TiO_2_ due to the possibility of the activation of an anatase–rutile heterojunction in the light range up to 400 nm. However, the obtained results indicate that PANI improved TiO_2_ activity in visible light at a wavelength above 420 nm.

To determine the mechanism of the photocatalytic degradation of the PANI–TiO_2_ nanocomposite, the photocatalytic activity analyses, in the presence of scavengers, were performed. The results are presented in Table 2. Benzoquinone (BQ), tert-butanol (*t*-BuOH)*,* silver nitrate (AgNO_3_), and ammonium oxalate (AO) were used as $O_{2}^{\cdot -}$ superoxide radical anions, ${\mathrm{OH}}^{\cdot }$ hydroxyl radicals, electron scavengers, and hole scavengers, respectively. The degradation rate constants without scavengers under UV-Vis irradiation served as references. The phenol degradation mechanism for bare TiO_2_ mainly resulted from the generation of electrons and superoxide radical anions, as evidenced by the decrease in the reaction rate constant. The scavenging of holes did not change the kinetics of phenol degradation. Recently, Pelaez et al. [55] reported that for TiO_2_ particles, the reactive oxygen species involved in the photodegradation reaction are strongly affected by the radiation range and pH of the reaction medium. At acidic conditions under UV-Vis light, mostly hydroxyl radicals are responsible for the degradation of the organic contaminants, whereas in the presence of Vis light electrons, superoxide radical anions play an essential role in the mechanism of xenobiotics degradation [55]. For PANI in the presence of BQ, t-BuOH, a slight decrease in the phenol degradation rate to 0.14 ± 0.01 µmol·h^−1^ was observed. In addition, for the AgNO_3_ scavenger, the phenol degradation rate decreased to 0.13 ± 0.01 µmol·h^−1^. However, in the presence of AO, the reaction rate even increased to 0.54 ± 0.03· µmol·h^−1^, respectively. Previously, for TiO_2_-based magnetic photocatalysts, the rate-enhancing effect of silver ions used as an electron scavenger was reported [56]. The photogenerated electrons can be trapped by Ag^+^ ions inhibiting the rate of the charge carriers recombination. Moreover, the enhanced photoactivity may result from the in situ formation of metallic species on the surface of the photocatalyst [56]. For the PANI–TiO_2_ nanocomposite, the addition of BQ and AgNO_3_ did not influence the phenol degradation rate constant, while the presence of *t*-BuOH caused a slight decrease in the constant rate. On the other hand, the addition of AO as a hole scavenger for PANI–TiO_2_ resulted in a decrease in phenol degradation. It suggests that for the PANI–TiO_2_ nanocomposite, photogenerated holes played a crucial role in the photodegradation mechanism.

**Figure 9:**
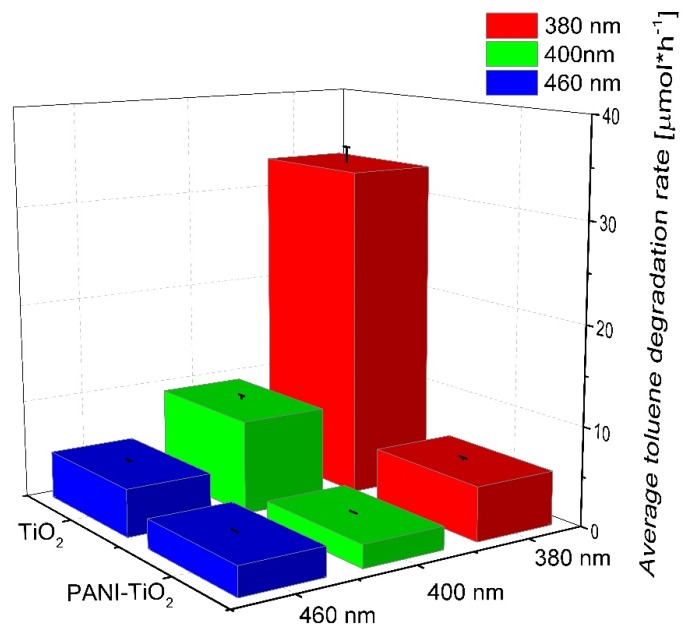
Photocatalytic activity of pure TiO_2_ and the PANI–TiO_2_ nanocomposite in the gas phase. Irradiation time: 3 h, toluene initial concentration: 200 ppm, photocatalyst loading: 15 mg.

Furthermore, the photocatalytic activity for pure TiO_2_ and the PANI–TiO_2_ nanocomposite in the gas phase was studied, and the results are presented in Figure 9. Pure TiO_2_ showed the highest photocatalytic activity under UV-Vis light. Almost complete degradation of toluene in the gas phase proceeded in 30 min and 3 h, using LEDs with λ_max_ = 380 nm and λ_max_ = 400 nm as an irradiation source, respectively. For the PANI–TiO_2_ nanocomposite under this condition, lower photocatalytic activity was obtained in comparison to pure TiO_2_. However, similar to the activity in the aqueous phase, when using irradiation of longer wavelengths (λ_max_ = 460 nm), an increase in the photocatalytic activity for PANI–TiO_2_ was observed (see also Figure S6). For hybrid photocatalyst irradiated using LEDs with λ_max_ = 460 nm, the toluene degradation rate was 2.94 ± 0.15 µmol·h^−1^. In comparison, for bare TiO_2_, the degradation rate was 4.80 ± 0.24 µmol·h^−1^ under the same conditions. Similar results were obtained in our previous study, on the degradation of toluene in the gas phase, over TiO_2_ modified with Pt, Cu, and Ag particles [53]. For LEDs with an emission peak maximum at 400 nm (UV-Vis), bare titanium(IV) oxide exhibited significantly higher activity in comparison to metal-modified TiO_2_. However, under visible light irradiation with λ_max_ = 460 nm, bare titanium(IV) oxide exhibited no activity, while metal-modified photocatalysts exhibited high activity resulted from the plasmonic excitations of metal nanoparticles on the TiO_2_ surface [53].

## 4. Photocatalytic Gypsum Plasters

**Figure 10:**
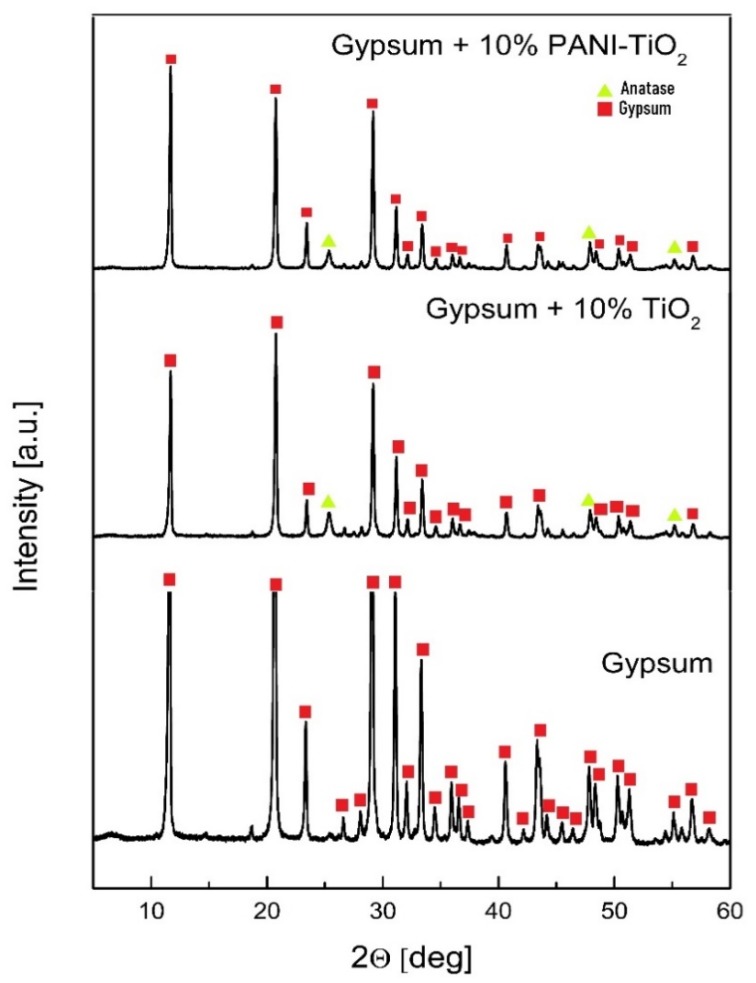
XRD patterns of gypsum and gypsum loaded with TiO_2_ or PANI–TiO_2_.

The photocatalytic gypsum plasters were prepared by mixing gypsum, the photocatalyst, and distilled water. The content of the photocatalyst (TiO_2_ and PANI–TiO_2_) was 10 wt % for all samples. The thickness of the plasters was 5 mm. The X-ray diffraction patterns for pure gypsum and gypsum modified with TiO_2_ and PANI–TiO_2_ are presented in Figure 10. The 2θ reflections for gypsum at 11.60°, 20.68°, 29.07°, and 31.07° corresponded to the followed gypsum structure planes: (020), (22-1), (24-1), and (021), respectively. For gypsum samples modified with TiO_2_ and PANI–TiO_2_, the 2θ reflections at 25.36° and 47.87° confirmed the TiO_2_ anatase (101) and (200) phase structures, respectively. The most intense signals for gypsum loaded with TiO_2_ and PANI–TiO_2_ at 11.69°, 20.77°, 29.15°, and 31.16° corresponded to gypsum (020), (12-1), (14-1), and (121) structure planes, respectively. Gypsum plaster with TiO_2_ contained 8% ± 2% of anatase and 98% ± 8% of the gypsum phase. Gypsum plaster modified with PANI-TiO_2_ had a lower anatase content than that modified with TiO_2_. The percentage content of phases in plasters loaded with PANI–TiO_2_ was 2.80% ± 0.11% and 97.2% ± 0.8% for anatase and gypsum, respectively.

The analysis of the contact angle for gypsum samples was carried out to determine changes in the surface properties of gypsum plaster after modification with a photocatalyst. Ethylene glycol was chosen as a liquid for the contact angle analysis. Based on the results presented in Table 3, it was observed that the addition of photocatalysts changed the hydrophilic/hydrophobic properties of the plaster surfaces. Modified gypsum plasters possessed a smaller contact angle, 20.6° ± 2.7° and 28.9° ± 0.6° for gypsum loaded with 10% of TiO_2_ and 10% of PANI–TiO_2,_ respectively than unmodified gypsum with a contact angle of 42.1° ± 1.9°. The presence of conjugated polymer on the TiO_2_ surface may reduce the hydrophilic character of titanium(IV) oxide particles. As a result, the greater contact angle was for PANI–TiO_2_-modified gypsum than for TiO_2_-modified gypsum due to the hydrophobic properties of the conjugated polymer.

**Figure 11:**
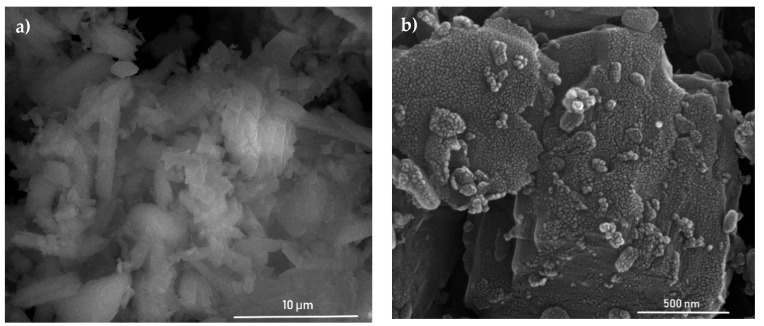
(**a**) SEM image of gypsum loaded with PANI–TiO_2_; (**b**) SEM image magnification of gypsum loaded with PANI–TiO_2_.

**Figure 12:**
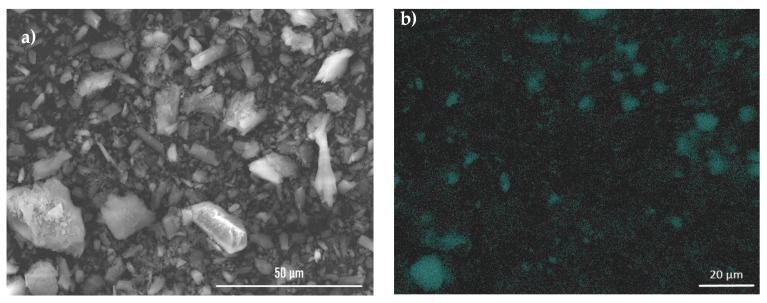
(**a**) SEM images of gypsum with PANI–TiO_2_; (**b**) EDS analysis for Ti (turquoise color).

Scanning electron microscopy analysis (see Figure 11 and Figure S7 in Supporting Materials) of gypsum loaded with PANI–TiO_2_ photocatalyst showed a coating of gypsum with a thin film of PANI–TiO_2_ composite. In addition, EDS analysis that was performed for gypsum with PANI–TiO_2_ confirmed this observation. Figure 12 shows that titania is distributed at the gypsum surface. The differences in the signal intensity for titanium in Figure 12b are because the surface of the analyzed sample is not perfectly smooth. The presence of PANI–TiO_2_ on the gypsum surface is in agreement with the previous results in the hydrophilic/hydrophobic properties of gypsum plaster after loading with the PANI–TiO_2_ nanocomposite. 

**Figure 13:**
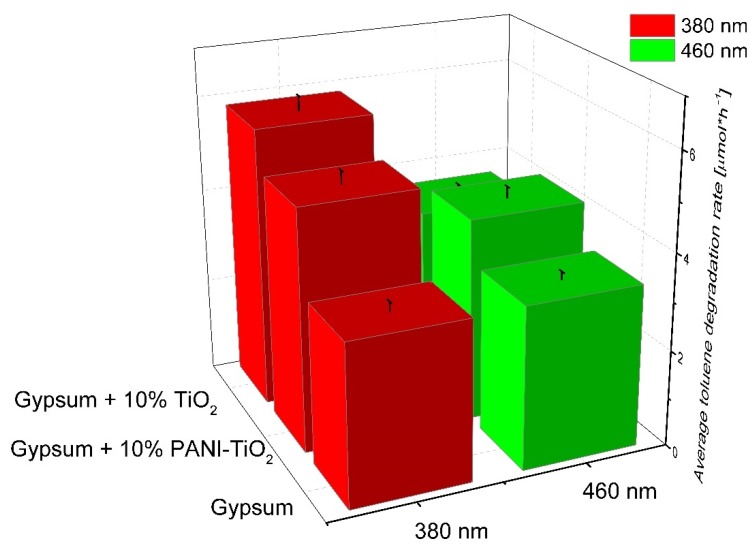
Toluene degradation in the presence of gypsum, gypsum loaded with TiO_2_, and gypsum modified with PANI–TiO_2._ Irradiation time: 3 h, toluene initial concentration: 200 pm; photocatalyst mass: 15 mg.

The model pollutant during the photocatalytic test was toluene, which was used at a concentration of 200 ppm in air. A summary of the toluene degradation constant rate under UV and visible light irradiation for gypsum samples is presented in Figure 13 and Figure S8. The gypsum plaster containing 10% of TiO_2_ had the highest photocatalytic activity in the gas phase reaction, as it was previously described in Section 3.2 for pure TiO_2_. The toluene degradation rates were 5.83 ± 0.29 µmol·h^−1^ for λ_max_ = 380 nm and 3.57 ± 0.18 µmol·h^−1^ for the 460 nm maximum irradiation wavelength, respectively. For gypsum plaster modified with PANI–TiO_2_, the rates were 5.02 ± 0.25 µmol·h^−1^ and 4.23 ± 0.21 µmol·h^−1^ for λ_max_ = 380 nm and λ_max_ = 460 nm, respectively. It is important to note that a decrease in the degradation constant rate for gypsum modified with TiO_2_ irradiated using LEDs with λ_max_ = 380 nm and LEDs with light emission at λ_max_ = 460 nm was observed. However, gypsum modified with 10% of PANI–TiO_2_ photocatalytic activity under visible light irradiation (λ_max_ = 460 nm) was higher than for LEDs emitting UV light (λ_max_ = 380 nm); see also Figure S8. The obtained results are in good agreement with those previously presented in this study for TiO_2_ and PANI–TiO_2_ results of photocatalytic activity in phenol and toluene degradation in aqueous and gaseous phases, respectively.

## 5. Discussion and Concluding Remarks 

In summary, the PANI–TiO_2_ nanocomposite was synthesized by an in situ oxidative chemical polymerization method. The photocatalytic activity of PANI–TiO_2_ was evaluated in aqueous and gas phases. The obtained results indicated that polyaniline absorbs the visible light with a maximum at 440 nm, and therefore, it can be an effective photosensitizer of TiO_2_ in a hybrid PANI–TiO_2_ nanocomposite. An analysis of photocatalytic activity was evaluated under polychromatic irradiation (UV-Vis, Vis > 400 nm, and Vis > 420 nm) in phenol degradation reaction in an aqueous phase and under selected narrow wavelengths emitted by LEDs in toluene degradation in the gas phase. It was also observed that the PANI/TiO_2_ photocatalytic activity mainly resulted from a direct electron–hole redox reaction over oxidation with reactive oxygen species.

**Figure 14:**
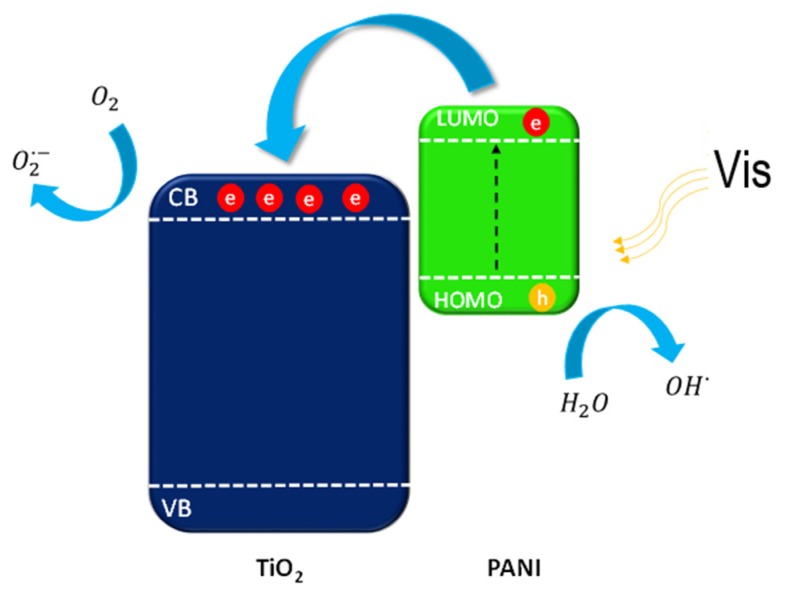
Mechanism of PANI–TiO_2_ nanocomposite excitation under visible light.

**Figure 15:**
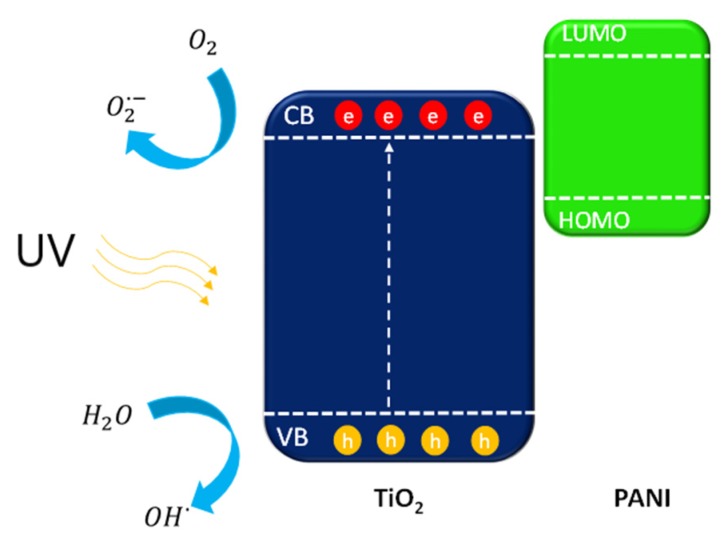
Mechanism of PANI–TIO_2_ nanocomposite excitation under UV light.

Based on the obtained results, the mechanism of photocatalytic degradation over PANI–TiO_2_ composites was proposed and presented in Figure 14 and Figure 15. The excitation of the semiconducting polymer as it was proposed by Heeger [57] may be described as:

(π-polymer)_n_ + hv → [{π-polymer}^+y^ + { π-polymer}^−y^]_n_, (4)

where y is a number of photoinduced electron–hole pairs.

Under the visible light irradiation, with a wavelength longer than 420 nm, polyaniline should act as a TiO_2_ photosensitizer. PANI can act as an efficient electron donor and hole transporter. The activation of PANI–TiO_2_ nanocomposites proceeded through the excitation of electrons from the ground state HOMO to the LUMO of polyaniline. Then, the excited electrons are transported into the TiO_2_ conduction band (CB) since the LUMO level of PANI is energetically higher than that of TiO_2_. Holes are accumulated on the HOMO orbital of PANI, which can react with compounds adsorbed on the nanocomposite surface. Our results indicate that positive charge carriers are the main species responsible for degradation over the PANI–TiO_2_ nanocomposite. An activation of titanium(IV) oxide-based photocatalysts under visible light irradiation may also be induced by incorporating nitrogen atoms into the TiO_2_ structure. Depending on the interstitial of substitutional doping, new energetic states between valence and conduction bands are created. For a hybrid composite of polyaniline and titanium(IV) oxide similarly, as for nitrogen-doped photocatalysts, strong interactions between titanium and nitrogen elements can occur, which may also lead to retarding the charge carriers recombination [58,59].

Under UV light, electrons from the TiO_2_ valance band (VB) are excited to the conduction band (CB). The electrons located on the CB of TiO_2_ were capture by oxygen (O_2_) dissolved in water to form $O_{2}^{\cdot -}$ or further react with H^+^ to yield ·OH. The ROS of h^+^, ·OH, and $O_{2}^{\cdot -}$ all participated in the photocatalytic degradation [59,60].

Nevertheless, the presence of polyaniline onto the TiO_2_ surface might block titanium(IV) oxide active centers, which resulted in a decrease of photocatalytic activity under UV light. The deterioration of the photocatalytic properties of the PANI–TiO_2_ nanocomposite under UV light may be a result of the thick PANI layer or fine TiO_2_ particle size; thus, further studies should consider the effect of the conjugated polymer content and the morphological properties of the TiO_2_ template on the structure and photocatalytic activity of PANI–TiO_2_ nanocomposites. Further study will be focused on the preparation and characterization of hybrid photocatalysts with different amounts of the conjugated polymer. We assume that island-type loading instead of the shell should enhance photocatalytic activity.

## Figures and Tables

**Table 1:** Crystalline structure and BET surface area of TiO_2_, PANI, and PANI–TiO_2_.

Sample Label	BET Surface Area (m^2^·g^−1^)	Average Crystallite Size (nm)			
		Anatase		Rutile	
		Size (nm)	Phase Content (%)	Size (nm)	Phase Content (%)
PANI	11	-	-	-	-
TiO_2_	55	18.2 ± 0.9	86.3 ± 0.3	25.4 ± 0.7	13.7 ± 0.2
PANI–TiO_2_	55	19.4 ± 0.9	81.3 ± 0.9	28.1 ± 0.7	12.9 ± 0.2

**Table 2:** Photocatalytic activity in a reaction of phenol degradation (Irradiation time: 60 min; photocatalysts loading: 2 g·dm^−3^; phenol initial concentration: 500 mg·dm^−3^; Irradiation source: UV-Vis, Vis > 400 nm, Vis > 420 nm).

Sample Label	Average Phenol Degradation Rate (µmol·h^−1^)						
	UV-Vis	Vis λ > 400 nm	Vis λ > 420 nm	Scavenger (UV-Vis)			
				BQ	t-BuOH	AgNO3	AO
PANI	0.27 ± 0.01	0.18 ± 0.01	0.12 ± 0.01	0.14 ± 0.01	0.14 ± 0.01	0.13 ± 0.01	0.54 ± 0.03
TiO2	4.63 ± 0.23	0.40 ± 0.02	0.12 ± 0.01	2.96 ± 0.15	1.16± 0.06	3.36 ± 0.17	4.92 ± 0.25
PANI–TiO2	2.01 ± 0.10	0.07 ± 0.01	0.26 ± 0.01	2.33 ± 0.12	2.02 ± 0.10	2.30 ± 0.11	1.40 ± 0.07

**Table 3:** The contact angle analysis for gypsum and gypsum modified with TiO_2_ or PANI–TiO_2_.

**Sample Label**	**Contact Angle (°)**	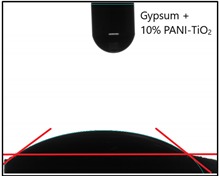
Gypsum	42.1 ± 1.9	
Gypsum + 10 wt % TiO_2_	20.6 ± 2.7	
Gypsum + 10 wt % PANI–TiO_2_	28.9 ± 0.6	

## Supplementary Materials

The following are available online at https://www.mdpi.com/1996-1944/13/7/1516/s1, Figure S1: The Tauc plots for TiO_2_, PANI and PANI-TiO_2_, Figure S2a–d: TEM microscopy analysis of PANI/TiO_2_ composite, Figure S3: Photocatalytic activity of TiO_2_, PANI and PANI-TiO_2_ in reaction of phenol degradation under UV-Vis light irradiation, Figure S4: Photocatalytic activity of TiO_2_, PANI and PANI-TiO_2_ in reaction of phenol degradation under Vis > 400 nm light irradiation, Figure S5: Photocatalytic activity of TiO_2_, PANI and PANI-TiO_2_ in reaction of phenol degradation under Vis > 420 nm light irradiation, Figure S6: Toluene degradation in time. The effect of irradiation source with maximum wavelength emission at 380 nm, 415 nm and 460 nm for a) TiO_2_ and b) PANI-TiO_2_ hybrid nanocomposite, Figure S7a–d: SEM images of gypsum surface modified with PANI-TiO_2_, Figure S8: Toluene degradation in time for gypsum, gypsum + 10% TiO_2_, and gypsum + 10% PANI-TiO_2_ using a) LEDs with a maximum wavelength emission at 380 nm, b) LEDs irradiation source with a maximum wavelength emision at 460 nm.
