# Supplementary material for: Hybrid TiO2–Polyaniline Photocatalysts and their Application in Building Gypsum Plasters
Source: Materials (Basel). 2020 Mar 26;13(7):1516. doi: 10.3390/ma13071516 (PMC7177723; doi:10.3390/ma13071516)
Supplement: Supplementary file 1 [file materials-13-01516-s001.pdf]

materials

Supplementary Materials

Hybrid TiO_2–Polyaniline Photocatalysts and their Application in Building Gypsum Plasters

Agnieszka Sulowska [1,*], Izabela Wysocka [1], Daniel Pelczarski [2], Jakub Karczewski [3] and Anna Zielińska-Jurek [1,*]

[1] Department of Process Engineering and Chemical Technology, Faculty of Chemistry, Gdansk University of Technology, 80-232 Gdansk, Poland; izabela.wysocka@pg.edu.pl

[2] Department of Physics of Electronic Phenomena, Faculty of Applied Physics and Mathematics, Gdansk University of Technology, 80-232 Gdansk, Poland; daniel.pelczarski@pg.edu.pl

[3] Department of Solid State Physics, Faculty of Applied Physics and Mathematics, Gdansk University of Technology, 80-232 Gdansk, Poland; jakub.karczewski@pg.edu.pl

* Correspondence: sulowska.as@gmail.com (A.S.); annjurek@pg.edu.pl (A.Z.-J.)

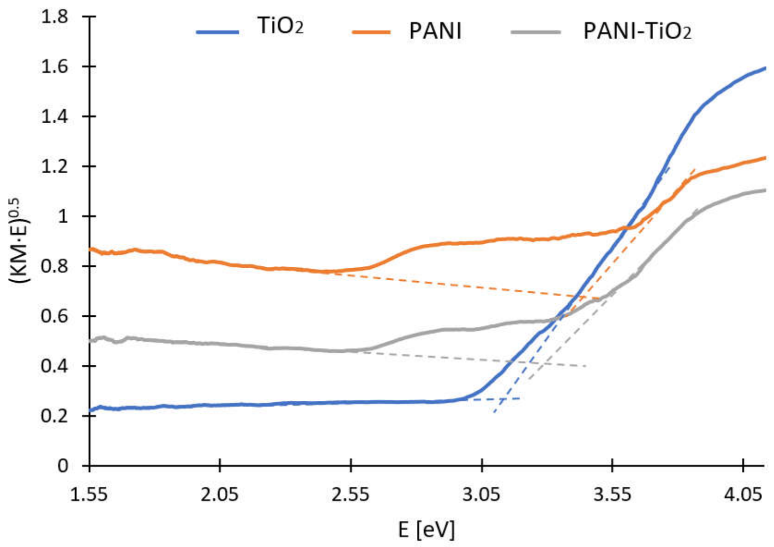

Figure S1. The Tauc plots for TiO_2, PANI and PANI-TiO_2.

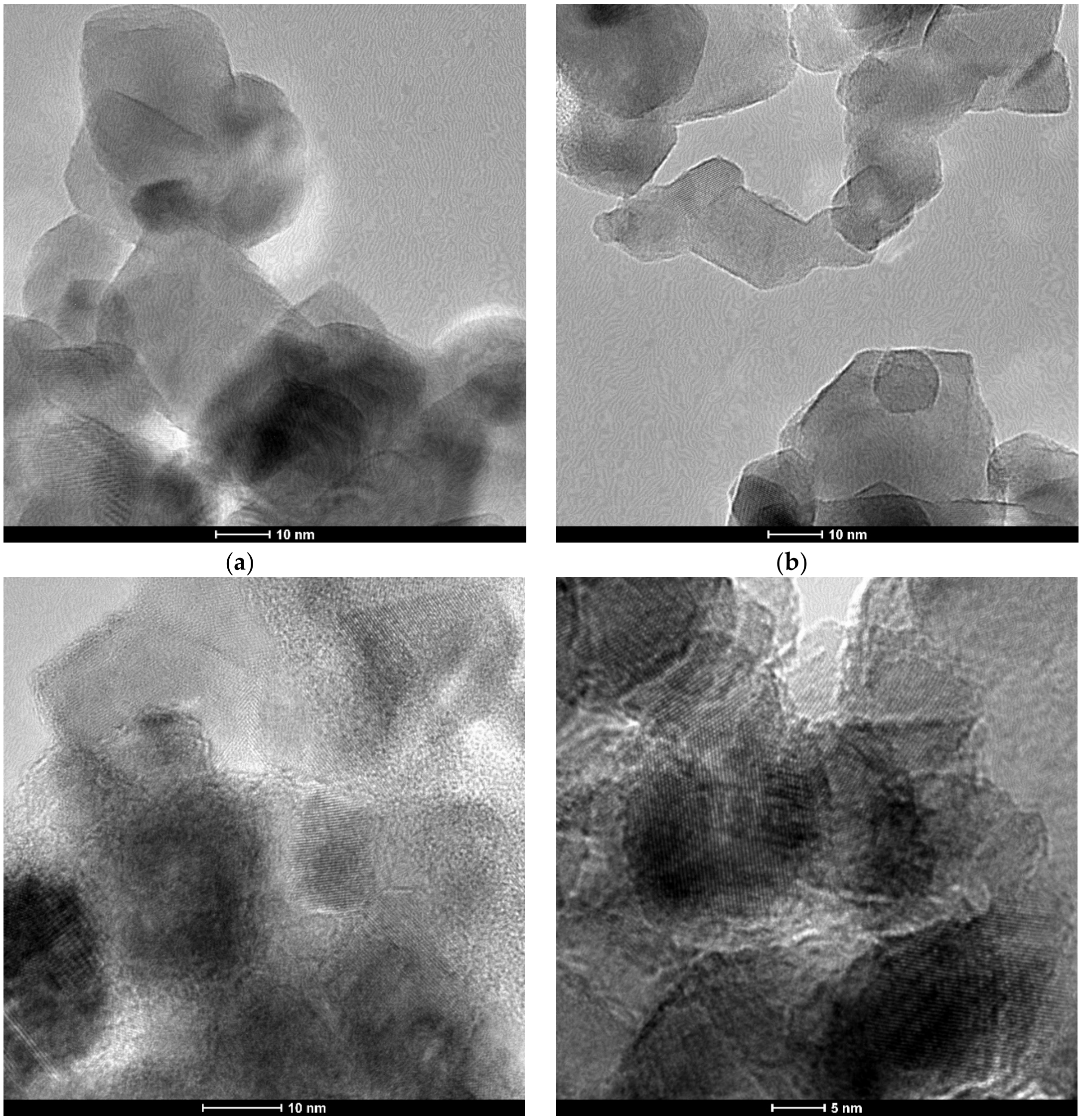

(**a**) (**b**)

(**c**) (**d**)

Figure 2. a–d: TEM microscopic images of PANI/TiO_2 composite.

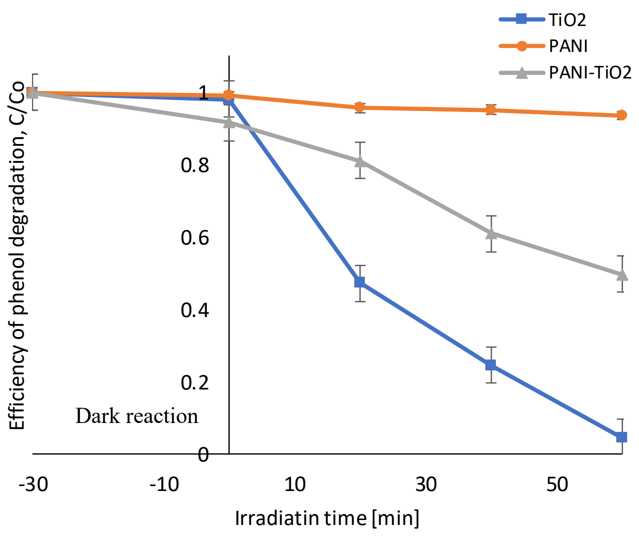

Figure S3. Photocatalytic activity of TiO_2, PANI and PANI-TiO_2 in reaction of phenol degradation under UV-Vis light irradiation.

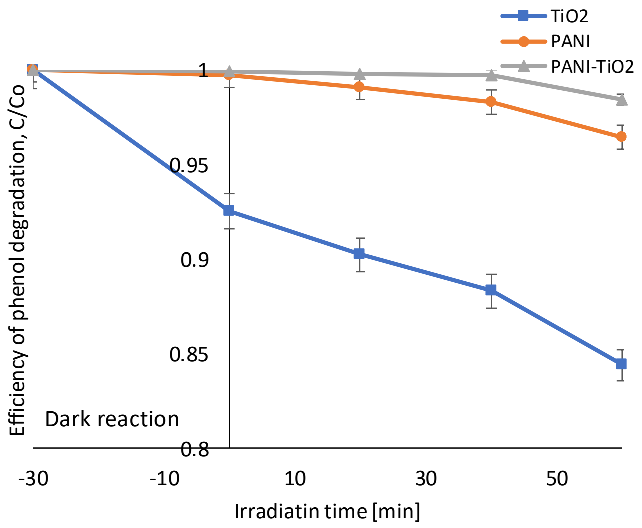

Figure S4. Photocatalytic activity of TiO_2, PANI and PANI-TiO_2 in reaction of phenol degradation under Vis > 400 nm light irradiation.

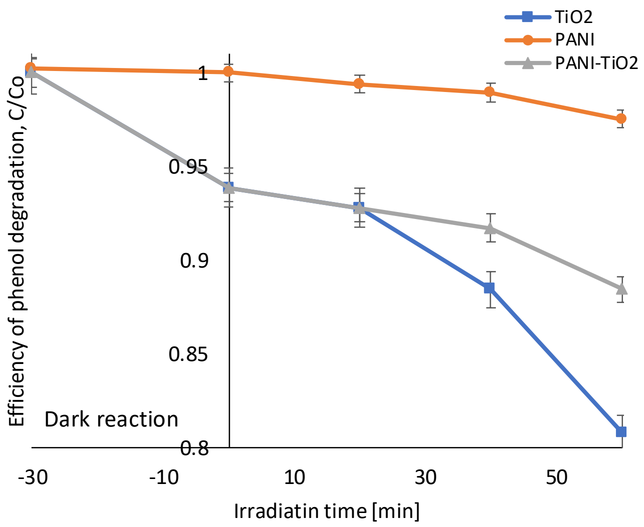

Figure S5. Photocatalytic activity of TiO_2, PANI and PANI-TiO_2 in reaction of phenol degradation under Vis > 420 nm light irradiation.

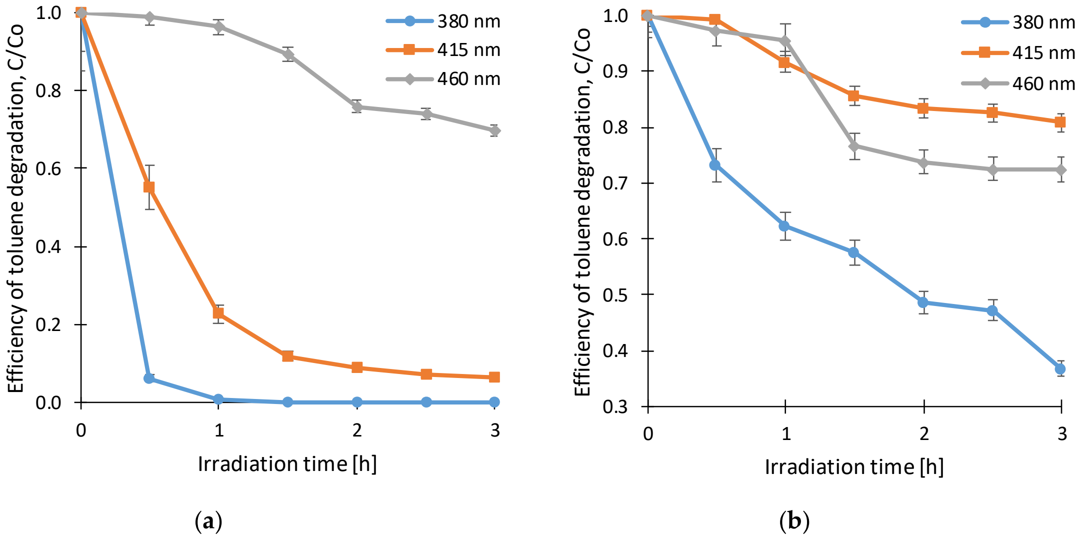

Figure S6. Toluene degradation in time. The effect of irradiation source with maximum wavelength emission at 380 nm, 415 nm and 460 nm for **a**) TiO_2 and **b**) PANI-TiO_2 hybrid nanocomposite.

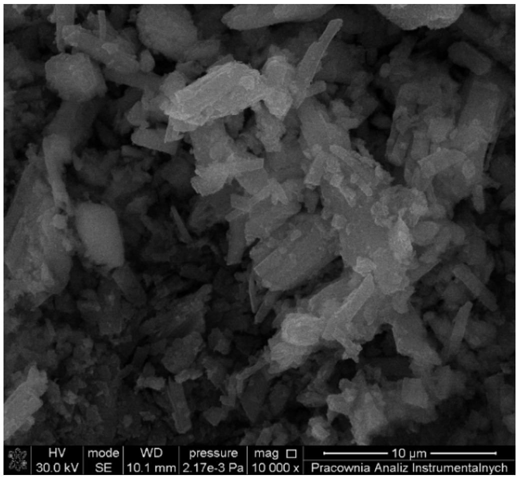

(**a**)

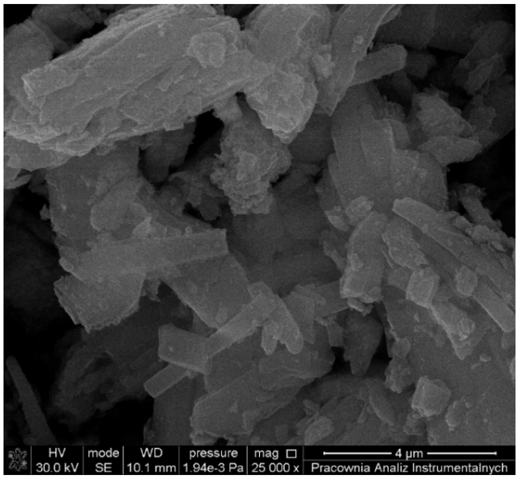

(**b**)

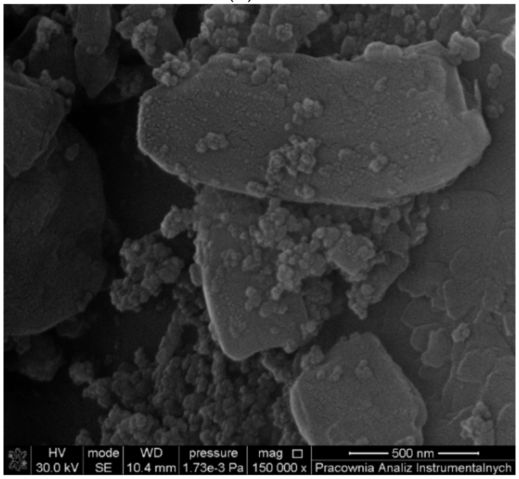

(**c**)

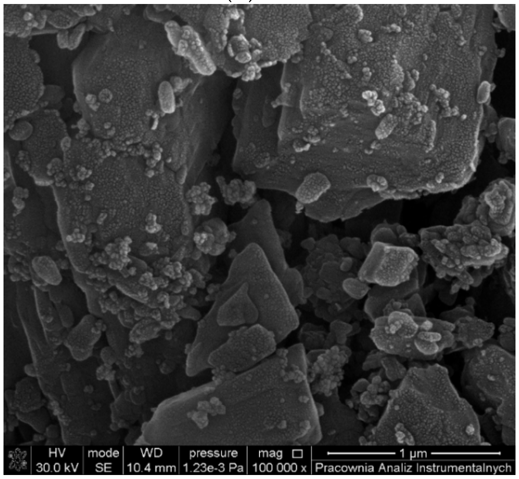

(**d**)

Figure 7. a–d: SEM images of gypsum surface modified with PANI-TiO_2.

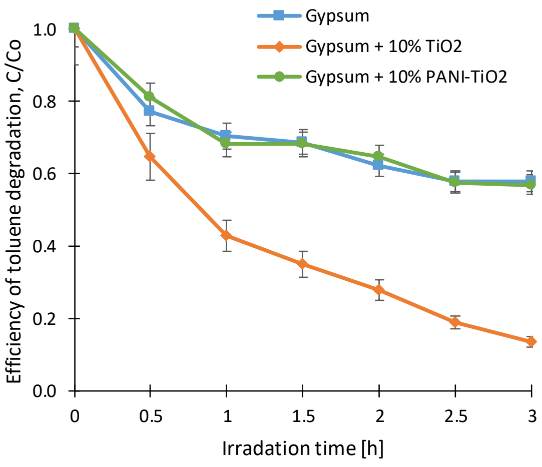

(**a**)

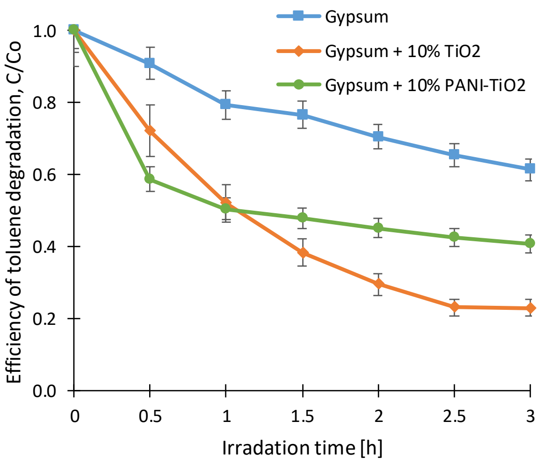

(**b**)

Figure S8. Toluene degradation in time for gypsum, gypsum + 10% TiO_2, and gypsum + 10% PANI-TiO_2 using **a**) LEDs with a maximum wavelength emission at 380 nm, **b**) LEDs irradiation source with a maximum wavelength emission at 460 nm.
